# Formative Evaluation of a Smartphone App for Monitoring Daily Meal Distribution and Food Selection in Adolescents: Acceptability and Usability Study

**DOI:** 10.2196/14778

**Published:** 2020-07-21

**Authors:** Billy Langlet, Christos Maramis, Christos Diou, Nikolaos Maglaveras, Petter Fagerberg, Rachel Heimeier, Irini Lekka, Anastasios Delopoulos, Ioannis Ioakimidis

**Affiliations:** 1 The Innovative Use of Mobile Phones to Promote Physical Activity and Nutrition Across the Lifespan Research Group Department of Biosciences and Nutrition Karolinska Institutet Huddinge Sweden; 2 Lab of Computing, Medical Informatics and Biomedical Imaging Technologies Department of Medicine Aristotle University of Thessaloniki Thessaloniki Greece; 3 Department of Electrical and Computer Engineering Aristotle University of Thessaloniki Thessaloniki Greece; 4 Internationella Engelska Gymnasiet Stockholm Sweden

**Keywords:** mHealth, eHealth, dietary behavior, lifestyle behavioral monitoring, lifestyle interventions, obesity, mobile phone, smartphone, weight management, overweight

## Abstract

**Background:**

Obesity interventions face the problem of weight regain after treatment as a result of low compliance. Mobile health (mHealth) technologies could potentially increase compliance and aid both health care providers and patients.

**Objective:**

This study aimed to evaluate the acceptability and usability and define system constraints of an mHealth system used to monitor dietary habits of adolescents in real life, as a first step in the development of a self-monitoring and lifestyle management system against adolescent obesity.

**Methods:**

We recruited 26 students from a high school in Stockholm, Sweden. After a 30-minute information meeting and 5-minute individual instruction on how to use an mHealth system (smartphone with app and two external sensors), participants used it for 2-3 weeks to objectively collect dietary habits. The app and sensors were used by the participants, without supervision, to record as many main meals and snacks as possible in real life. Feasibility was assessed following the “mHealth evidence reporting and assessment checklist,” and usability was assessed by questionnaires. Compliance was estimated based on system use, where a registration frequency of 3 main meals (breakfast, lunch, and dinner) per day for the period of the experiment, constituted 100% compliance.

**Results:**

Participants included in the analysis had a mean age of 16.8 years (SD 0.7 years) and BMI of 21.9 kg/m2 (SD 4.1 kg/m2). Due to deviations from study instructions, 2 participants were excluded from the analysis. During the study, 6 participants required additional information on system use. The system received a ‘Good’ grade (77.1 of 100 points) on the System Usability Scale, with most participants reporting that they were comfortable using the smartphone app. Participants expressed a willingness to use the app mostly at home, but also at school; most of their improvement suggestions concerned design choices for the app. Of all main meals, the registration frequency increased from 70% the first week to 76% the second week. Participants reported that 40% of the registered meals were home-prepared, while 34% of the reported drinks contained sugar. On average, breakfasts took place at 8:30 AM (from 5:00 AM to 2:00 PM), lunches took place at 12:15 PM (from 10:15 AM to 6:15 PM), and dinners took place at 7:30 PM (from 3:00 PM to 11:45 PM). When comparing meal occurrence during weekdays vs weekends, breakfasts and lunches were eaten 3 hours later during weekends, while dinner timing was unaffected.

**Conclusions:**

From an infrastructural and functional perspective, system use was feasible in the current context. The smartphone app appears to have high acceptability and usability in high school students, which are the intended end-users. The system appears promising as a relatively low-effort method to provide real-life dietary habit measurements associated with overweight and obesity risk.

## Introduction

Obesity prevalence in children and adolescents between the ages of 5 and 19 increased 8-9 times between 1975 and 2016 [1]. Obesity, in turn, increases the risk of physical and mental health complications such as osteoarthritis, type 2 diabetes mellitus, and coronary heart disease [2], along with depression [3]. In addition, a lifetime of obesity increases the risk of disability and loss of income [4].

Comprehensive lifestyle interventions, targeting diet, physical activity, and lifestyle elements in tandem, are recommended by the American Heart Association, American Colleague of Cardiology, and The Obesity Society [5] as a means of accomplishing long-term weight loss and weight maintenance in adults. Indeed, studies have shown that lifestyle interventions can promote moderate weight reduction with low risk of adverse effects [6,7], even in children between the ages of 0 and 18 years, as reported in a recent Cochrane review [8]. However, most lifestyle interventions face the problems of low compliance and high drop-out rates, with interventions often reporting weight regains of almost 50% at the 1-year follow-up mark[9,10]. In primary care, while it seems possible for obesity treatment to be “relatively cost-effective” in the short term [11], long-term weight maintenance requires intensive management in the form of frequent visits and multiple contact hours [12,13]. Others have identified additional means for improving the effectiveness of interventions, including frequent and open communication among patients and health professionals, realistic behavioral goal setting, and continuous patient monitoring [14,15]. In practice though, the inherent time and monetary cost of involved health monitoring often shift the burden to the individuals, requiring them to self-monitor and self-manage their behaviors for longer periods of time [16]. However, this further increases the required effort from the side of the individual, potentially reducing compliance and increasing drop-outs.

To meet this challenge, novel mobile health (mHealth) tools can be developed to assist both patients and health professionals. Indeed, with the accessibility and use of smartphones greatly increasing [17], smartphones are a cheap, accessible option for implementing interventions. This is even more pronounced for interventions targeting children and adolescents since they are early adopters of new technology [18]. Additionally, smartphones (and other wearable electronic devices) create the opportunity for collecting information that may be difficult for users to self-report, such as exact times, locations, and types of behaviors [19], resulting in increased user compliance with smartphone-based interventions [19,20].

Current efforts for the development of large-scale, non-invasive dietary behavioral monitoring systems appropriate for use by children and adolescents in challenging real-life environments, like school, are limited [21]. However, novel methodologies are constantly being tested [22], including our own efforts for collection of large-scale Big Health Data about the dietary and physical activity habits of school children [23]. However, such large-scale deployments require conscious efforts for preliminary testing outside the lab, in the target use environment, to identify potential use constraints and optimize the deployed methodologies.

On a commercial level, smartphone apps claiming to promote weight loss have seen a huge increase recently, but their alleged effectiveness lacks support by large-scale, long-term clinical studies [24]. Meanwhile, the number of mHealth self-management programs against obesity constantly increases, but due to mHealth being in its infancy, evidence for their effectiveness is sparse [25]. Recent meta-analyses have concluded that mHealth interventions performed similarly, if not better, then traditional non-mHealth–based obesity interventions [26-28] but emphasized the heterogeneity in the quality of the existing evidence base. Realizing the lack of consensus on mHealth reports, the World Health Organization has developed a framework for what and how to report on the various components of an mHealth intervention, called the “mHealth evidence reporting and assessment checklist” (mERA), aimed at improving the quality of the existing evidence [25]. Once the risk population has been identified, interventions should identify ways to reach the population in question, performing a formative evaluation, focusing on gathering functional requirements, and developing and testing the technology in a descriptive way [29]. Evaluating the effectiveness of mHealth in the intended population requires the evaluation of at least three components: usage (objective), acceptability (subjective), and feasibility of implementation [25].

In this study, the aim was to evaluate the acceptability, usability, and constraints of a newly developed mHealth system used for monitoring eating behaviors and dietary habits of adolescents in real life, as a first step for the development of a more comprehensive self-monitoring and lifestyle management system. The evaluation of the system was conducted on two levels: subjective self-reports by users and objective compliance estimations of the study protocol (eg, frequency of registering eating events). The system’s evaluation followed the mERA checklist for a more transparent report of its feasibility and usability. At its core, the developed system employed a smartphone to collect data from a smartphone app and two additional sensors, and all data were automatically exported to a centralized data collection platform where the data were analyzed and results collected. The smartphone app allowed participants to self-report eating events throughout the day, while the two additional sensor modules enabled collection of additional objective eating behavior data. The development of these modules, a food scale, and a chewing sensor has been previously described elsewhere [30], and their use was complementary and fully integrated with the system described herein. Finally, this report presents population-level data on dietary habits associated with the development and maintenance of adolescent obesity (ie, frequency of meals consumed in restaurants, frequency of sugary beverage consumption, and analysis of meal timing across days during weeks vs weekends). The detailed analyses of these datasets provide additional evidence of the usefulness of the system, setting the basis for the development of future interventions against obesity in the targeted population.

## Methods

### Subjects

There were no specific inclusion or exclusion criteria, and all students from 6 classes of the collaborating school were informed of the study. The classes were selected together with the school administration based on their end-of-the-year scheduled obligations and their prior participation in a past relevant study [31]. With 26 system units (a smartphone together with a digital food scale and chewing sensor) available, a first-come-first-serve approach was used for recruitment. Participants 18 years or older provided written consent, while younger participants provided written assent together with written consent by their legal guardians. The forms to be signed were provided to the students after an informational meeting, where students could ask for clarifications regarding the protocol of the study. The researchers returned to the school on two later occasions to collect the signed forms. Ethical approval was provided by the Stockholm Regional Ethics Board (D.nr.: 2015/1824-31), and the presented practices followed the Declaration of Helsinki’s guidelines for human research. Participants who completed the study received cinema tickets as compensation for their participation. All the participating students had previously participated in a study including lunch recordings in a school cafeteria, which followed a protocol described elsewhere [31].

### Experimental Design

During the study, the subjects participated in a data collection action lasting between 2 and 3 weeks that took place towards the end of the school year. During this period (varying for each student due to their individualized end-of-year obligations), the students were asked to register all their weekday and weekend meals. The basic meal registration was done using a smartphone, and participants could contribute additional eating behavior data by using either the food scale or chewing sensor.

### Study Protocol

The protocol of the study was uncontrolled by design, aiming to capture the true dietary habits of the participants, diminishing the required effort on their part. All textual components of the system were in English, in agreement with the teaching language in the selected school. The study began by participants receiving information on system use and study protocol twice: first in a group meeting lasting for 30 minutes and then on an individual level for approximately 5 minutes. During the individual meeting, the students received information on system use, went through all the screens and options of the mobile app, and were handed the smartphone and complementary devices, also signaling the initiation of the data collection. The participants were requested to register all their meals as they happened (not retrospectively) during the study period using the provided smartphone app and were also asked to use the provided food scale to collect additional information for main meals (breakfast, lunch, and dinner) and the chewing sensor to collect information for main meals and snacks. Depending on the students’ school schedule and their individual school obligations for the end of the school year, the study duration ranged from 2 to 3 consecutive weeks, after individual arrangements with their supervising teachers. A researcher was available to provide technical and protocol support to the students from Monday to Friday (school days) throughout the study. After the predefined end of the data collection period per student, the participants returned the devices to the researchers and completed the system usability and user experience questionnaires.

### Devices and Smartphone App

**Figure 1:**
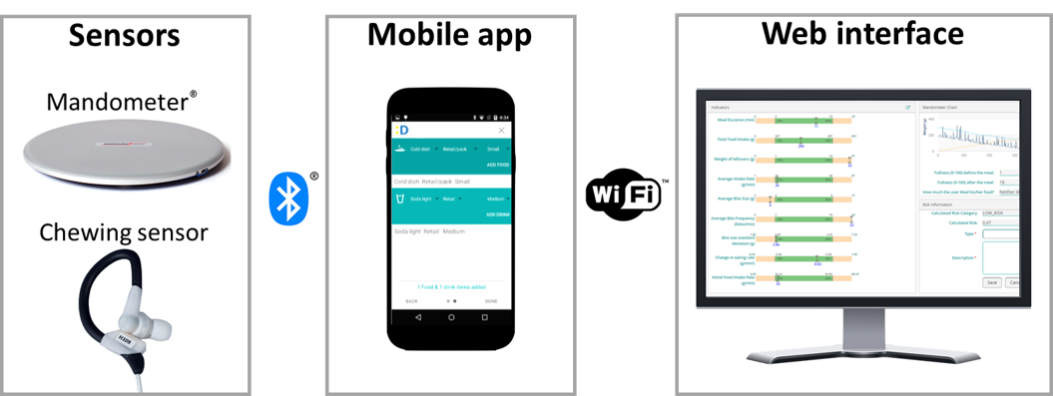
The system consists of a digital food scale, chewing sensor, smartphone app, and web app.

For the duration of the experiment, all participants were provided with an Android smartphone with the required smartphone app installed, as well as a food scale and chewing sensor [30]. We have previously published the methodology and development processes for the integrated sensors (for a system overview, see Figure 1). In summary, the user was presented with 3 options for registering any eating or drinking occurrence: self-register the event using only the mobile app, use the mobile app and food scale to record their plated meals through continuous registration of the food weight remaining on their plate [32], or wear the chewing sensor to record their eating or drinking event automatically through integrated photoplethysmography and acoustic analysis [28]. The provided smartphones did not have SIM cards, dictating the use of Wi-Fi networks (at school or home) to transmit the collected data. All other nonstudy-related smartphone apps and functions (ie, GPS) were turned off. Smartphones were provided to the students by the study because the high prevalence of iOS devices in Sweden [33] would prohibit the participation of otherwise motivated students, and we strived to homogenize the user experience for all the students, providing similar phone models to exclude parameters like varying device performance, custom device interfaces, and differing battery consumption rates.

**Figure 2:**
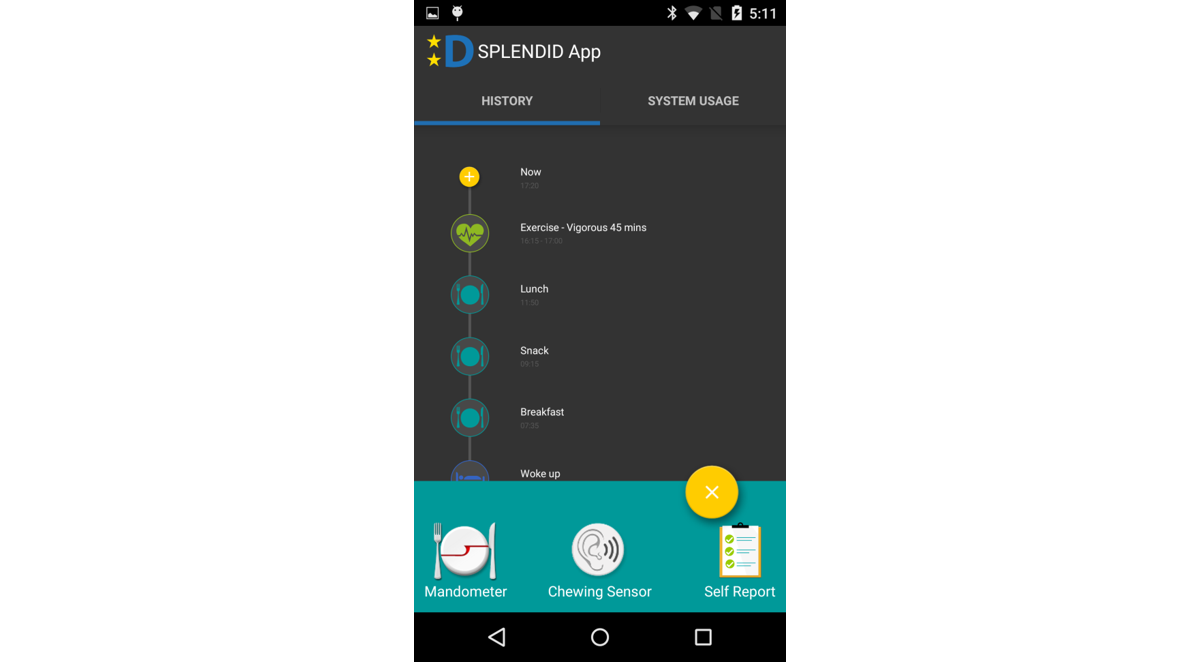
Main screen of the smartphone app.

Registering eating events was supported through a custom developed smartphone app available for the Android operating system. The app allowed registering either a meal or a drink separately (Figure 2) or registering a meal and adding complementary drinks. In all cases, irrespective of the means used, a timestamp was automatically generated and saved by the system. In the case of the chewing sensor, which automatically detected eating and drinking events, the system presented the user with a persistent notification asking for verification of the detection and allowing the user to fill in additional event information. For every meal, the participants were prompted to answer two additional questions: meal type (breakfast, lunch, dinner, or snack) and where the food was prepared or bought (home, retail store, or restaurant). The maximum number of registerable main meals (breakfast, lunch, and dinner, as characterized by the users) for each participant was 3 per day, with all the remaining eating occasions automatically registered as snacks. Participants could not use multiple methods to register a main meal (ie, self-registration, food scale, and chewing sensor). Once a meal had been registered with one method, it could not be registered again using a different method. However, the users were free to register less than 3 main meals per day, in cases where they skipped or forgot to register some main meals. During drink registration, the participants had the option to include additional information about the type of the consumed drink (sugary drink, coffee/tea, dairy/milk, or water). When the smartphone was connected to Wi-Fi, the registered meals were automatically uploaded online to a study server, allowing the researchers to supervise the progress of the study in real-time using a dedicated web interface. All the communications between the browser and server were encrypted. In conclusion, the system compiled an integrated matrix of meal and drinking event timestamps, irrespective of the registration method. The users were instructed to use the smartphone app and provided weighing scale throughout the day and charge them overnight. Battery concerns dictated the use of the chewing sensor only during the after-school hours (ie, between 5:00 pm and 11:00 pm) for registration of drinks and meals, with the device also being recharged overnight. The participants did not have to charge the provided scales, since a full charge lasted more than 3 weeks.

### System Usability and User Experience Questionnaires

System usability was reported using the System Usability Questionnaire (SUS) [34], and user experience was estimated based on a custom questionnaire developed in discussion with adolescents from the same school in a previous study. In the user experience questionnaire, the smartphone app, digital food scale, and chewing sensor were rated on how comfortable they were to use and if the users perceived that the device use affected their usual behavior, as well as if the technology was potentially usable ‘in school’, ‘at home’, or ‘outside’ on 10-point Likert scales, ranging from 0 for ‘Completely disagree’ to 9 for ‘Completely agree’. As part of the same questionnaire, participants also provided free-text answers on what they would improve for the system and its individual components (smartphone app, digital food scale, and chewing sensor). The questionnaire also contained a free-text question on the price of the system (smartphone app, food scale, chewing sensor, excluding server use), which was compared with the suggested pricing by the developers and manufacturers. The SUS has been validated on multiple occasions [35], while the custom behavior change and comfort questionnaire was previously used in similar studies [31].

### Data Analysis

The presented figures were generated using R 3.5.1. Feasibility is presented based on the mERA guidelines, with the checklist available in Multimedia Appendix 1 [25]. Estimation of compliance was made by dividing the number of registered main meals with the maximum number of main meals each participant was expected to register, based on the functionality of the provided system and the provided study instructions (ie, 3 main meals per day: one breakfast, one lunch, and one dinner). The formula for daily participant compliance was compliance = registered main meals/3. Food type frequency and meal timing are presented on a population level, with daily meal distribution presented in 24-hour day cycles starting at 4:00 am (ie, meals reported between 00:00 am and 3:59 am were grouped with meals registered during the previous calendar day) following a previously used practice [36]. All the values presented in the text are mean (SD).

## Results

### Subjects

Of the 26 adolescents that participated in the study, the datasets provided by 2 participants were excluded from the analysis due to significant deviations from the study protocol, such as device misuse (ie, prohibited use of the phone video camera in school, which is not supported by the current ethical application) and manually unlocking and using additional mobile features (eg, online mobile games). This resulted in 24 of the participants (24/26, 92%) being included in the analyzed study sample (Table 1).

**Table 1:** Group characteristics.

Characteristics	Total (n=24), mean (SD)	Men (n=7), mean (SD)	Women (n=17), mean (SD)
Age (years)	16.8 (0.7)	17.2 (0.5)	16.6 (0.7)
Weight (kg)	62.2 (14.4)	75.7 (10.1)	56.6 (12.1)
Height (cm)	168.1 (10.3)	181.3 (6.8)	162.7 (5.3)
BMI (kg/m^2^)	21.9 (4.1)	23.3 (4.7)	21.3 (3.8)

### Feasibility

Analyzing the system feasibility based on the mERA checklist [25], in the current study, phones without SIM cards and with locked hosting features were provided to all participants to ensure user interaction was restricted to the research app and to better manage confidentiality issues, which was a sensitive issue due to data collection by adolescents and especially in the school environment. Conceptually however, since all participants owned a smartphone, the system could easily have been used on the participants’ own phones, but that would require that the smartphone app was developed for multiple operating systems and not only Android. The software ran well and displayed properly on Android smartphones with 1.0 GB RAM, 8.0 GB ROM, and at least a 1280x720 HD display. All students had access to Wi-Fi both at home and at school. The battery lives of the smartphone and food scale allowed recordings throughout the day, while battery limitations restricted chewing sensor recordings to 6 hours per day. No protocol adaptations were required during the study as a result of unexpected complications. In total, 6 participants required additional information from researchers on specifics of system use, and 2 food scales had to be replaced during the experiment due to equipment malfunction (excessive battery drainage). All malfunctions were identified and addressed within 1 day. The most time-sensitive and laborious period for the researcher during the study was at the initiation, when information was provided to participants while handing out smartphones and devices. The remainder of the study required limited additional effort, mostly in the form of short interactions with individual students to answer follow-up questions or when data from the web platform indicated equipment malfunction or low compliance. The companies developing the sensors suggested a single purchase cost of €200 for the system, including both sensors and the app. Meanwhile, the price suggested by participants for the system varied greatly, with a mean single purchase cost of €67 (ranging from €14 to €236) or a monthly cost of €6 (ranging from €0 to €19).

### System Usability and User Experience

Participants rated the system usability between 47.5 and 97.5, with a mean value of 77.1, which corresponds to a grade B ‘Good’ rating, on the SUS. No adverse events were reported to researchers during the experiments or to school personnel after the experiment. The smartphone app and chewing sensor received the highest and lowest ratings for comfort, respectively. For perceived usability, all system components (smartphone app, food scale, and chewing sensor) scored highest at home and lowest outside home (Table 2).

Suggestions for improvements of the smartphone app fell into 4 categories: (1) 5 individuals suggested making it more responsive or faster, (2) 4 wanted a brighter color theme, (3) 3 wanted the function to retrospectively add meals, and (4) 3 requested better app interfaces, without specifying what was lacking.

**Table 2:** Perceived usability and acceptability of the smartphone app and devices, answered using 10-point Likert scales, ranging from 0 (completely disagree) to 9 (completely agree).

Statements	App, mean (SD)	Food scale, mean (SD)	Chewing sensor, mean (SD)
I felt comfortable using the …	7.3 (1.8)	6.4 (2.3)	3.9 (2.8)
Perceived behavioral change from using …	4.0 (2.2)	3.6 (2.6)	3.9 (2.5)
Potential for use of ... in school	6.3 (2.8)	5.4 (3.2)	3.5 (2.7)
Potential for use of … at home	7.1 (2.3)	7.1 (2.5)	4.6 (3.1)
Potential for use of … outside	5.4 (2.5)	3.8 (2.3)	3.1 (2.6)

### Registering the Frequency of Main Meals

**Figure 3:**
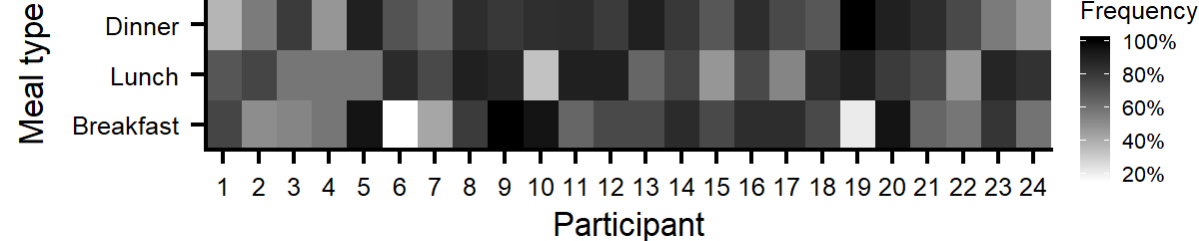
Frequency of meal registration per participant throughout the study, ranging from 0% to 100% per day for each main meal type.

On average, participants collected data for 18.4 days (SD 1.3 days). On average, each participant registered 63.3 meals (SD 18.0 meals), of which 39.5 meals (SD 6.3 meals) were main meals (breakfast, lunch, and dinner) and 23.8 meals (SD 14.9 meals) were snacks. Of the 39.5 main meals, 12.6 meals (SD 4.0 meals) were breakfasts, 13.2 meals (SD 3.0 meals) were lunches, and 13.7 meals (SD 3.3 meals) were dinners. In 51% (775/1520) of the overall reported meals, participants used a sensor to provide additional data. Of these meals, 87% (674/775) were made using the food scale, and the remaining meals (101/775) were registered with the chewing sensor. The average estimated compliance per participant was 73% (2.2/3 main meals per day) across all study days. To measure changes in compliance over time, only data from the first 2 weeks of system use were compared, during which the mean number of registered meals per participant was 50.2 (SD 13.9 main meals). On the group level, the number of registered meals from the first week was 573, of which 355 were main meals and 218 were snacks. Meanwhile, during the second week, 632 meals were registered, of which 384 were main meals and 248 were snacks. This shows an increased registering frequency from 70% (355/504) to 76% (384/504) of the 3 expected main meals per day from week one to week two. There were large variations between individuals in the type of main meals that were registered in the study (Figure 3).

### Population Food Selection Frequency

Regarding food preparation, 40% (608/1520) of the registered meals were ranked as home-cooked, 46% (699/1520) as purchased in food stores, and 14% (213/1520) as consumed in restaurants. Regarding drink types, 34% (314/924) of the registered drinks were sugary drinks, 15% (139/924) were dairy-based, 34% (314/924) were water, and 17% (157/924) were tea or coffee.

### Population Mealtime Distribution

**Figure 4:**
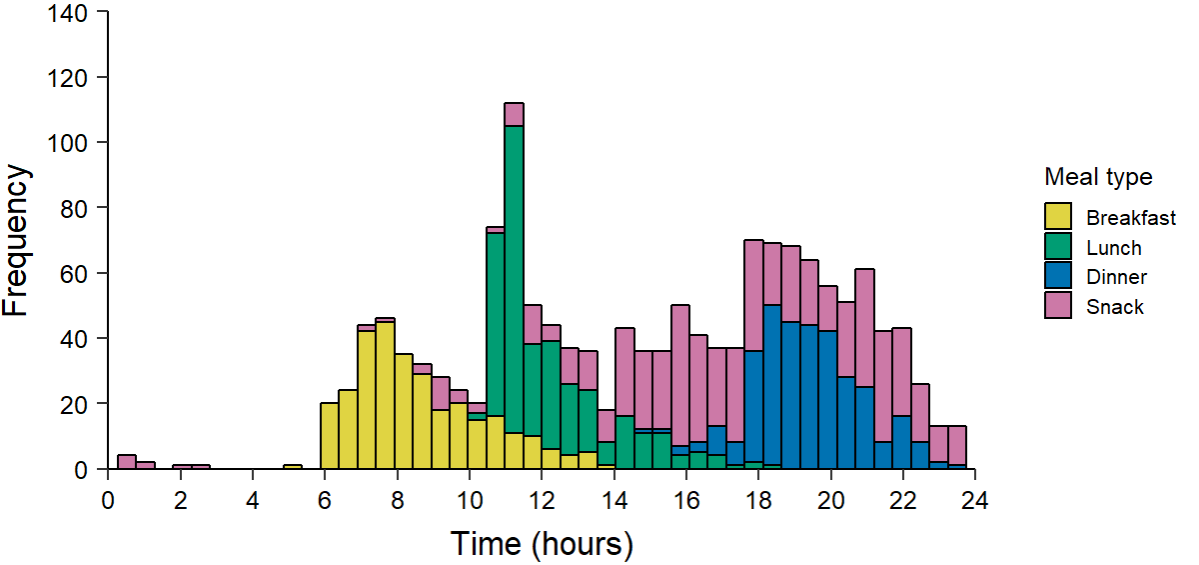
Histogram depicting the meal distribution of breakfast, lunch, dinner, and snacks across the day for all registered meals. Registration was done by 24 participants for a period of 2-3 weeks.

On average, breakfast took place at around 8:30 am, ranging from 5:00 am to 2:00 pm. On average, lunch took place around 12:15 pm, ranging from 10:15 am to 6:15 pm. Meanwhile, on average, dinners took place around 7:30 pm, ranging from 3:00 pm to 11:45 pm (Figure 4).

### Distribution of Meals Across Weekdays and Weekends

**Figure 5:**
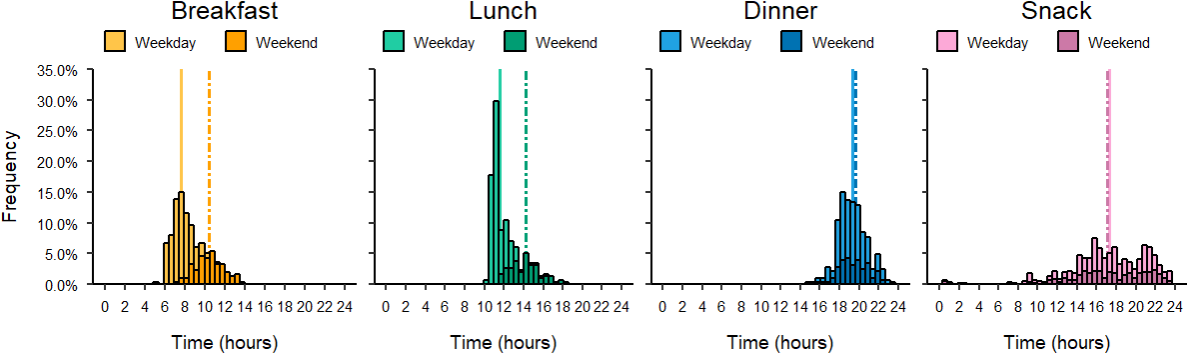
Histogram showing the distribution of all registered meals. Vertical lines in the graphs show the mean values of weekday (solid line) and weekend (dashed line) meal occasions.

The majority of breakfasts were eaten earlier during weekdays compared to weekends, with a difference of 2 hours 49 minutes between the mean value for weekday and weekend breakfasts. Similarly, lunches were generally eaten earlier during weekdays than during weekends, with a difference of 2 hours 44 minutes. Dinners were eaten slightly earlier during weekdays than during weekends, with a difference of 17 minutes. There was a slight difference between the time (14 minutes) of reported snacks between weekends and weekdays, with snacking overall being spread out throughout the monitored days (Figure 5).

## Discussion

This study evaluated the perceived acceptability and usability by adolescent participants of a system composed of a smartphone app and two eating behavior sensors, while identifying system constraints for future large-scale deployments. The app was well received by the participants, while the sensors received lower scores of usability and acceptability. From a functional and infrastructural perspective, the system had easy-to-meet requirements, was easy to use, and had high scalability. The usefulness of the system also appeared high, with its ability to collect data on dietary behaviors associated with increased risk of obesity (ie, sugary beverage consumption, restaurant meals, and unstructured meal patterns). From a functional perspective, the technical requirements of the smartphone app were low, including battery consumption, ensuring that the presented app runs on most modern smartphones, with 99% of Swedish 16–25-year-olds using smartphones regularly and a little less than 95% of devices in Sweden fulfilling the 1 GB RAM and 8 GB ROM requirement [33]. In addition, in Sweden, smartphone coverage and accessibility to Wi-Fi and energy outlets are high, making a screening protocol, such as the one explored here, highly feasible from an infrastructural perspective [17], ensuring the scalability of the system. Concerning the system’s suggested cost, a discrepancy existed between the system creators and the end users, which is expected due to the targeted age group. For the researcher, little work was required to ensure proper use of the smartphone app and devices, with only one-fourth of participants requiring additional information on specific functions of the system despite the short duration of the initial information meetings. The dedicated research platform enabled real-time data inspection, which made the identification of malfunctioning devices easy and ensured high data retention throughout the study. Participants found the system easy to use, with the total system receiving a high system usability score. Some participants felt that the smartphone app was a bit slow and unresponsive at times, which could potentially have resulted in lower compliance, but this is not immediately evident in the output dataset. A recent study identified personalization options as a strategy to increase interaction and compliance [37], a potential answer to our user requests (7/24) for improved interfaces. Finally, the possibility of retrospectively registering meals is a feature often seen in comparable apps. Future versions of our system might integrate this functionality to reduce the risk of the user forgetting to report meals as they occur, an issue that might have affected the reported outcomes of this study. However, the addition of retrospective meal reporting has the potential to introduce additional recall bias, resulting in decreased accuracy for the collected measurements [38].

The high frequency of meal registration per individual makes us confident that the mealtime distribution results are representative of the population’s actual mealtime distribution. In addition, the increased number of reported meals from week one to week two suggests that a familiarization period, or more training in device use, might increase registering frequency. In a study with a similar sample population (ie, conducted in adolescents in Sweden) that employed a 7-day food record, the number of registered meals was 18% higher than in the current study [39], which might be related to the lack of functionality for retrospective meal reporting in our own system. However, due to the allowance of recall data and differences in main meal definitions (primarily breakfast) and methodology (recollection vs real-time registering) as well as a potentially higher socioeconomic status in the current sample, it is unlikely that these two studies are directly comparable. Future studies should therefore aim to compare the methods employed here with 24-hour/day automatic meal registration methods, such as the ones developed by Sazonov et al [40], Sun et al [41], and Kyritsis et al [42], which have the potential to remove recall and social desirability bias (eg, where individuals only register products that are viewed favorably by others) but may introduce other biases, such as comfort of use. This will be even more relevant once the chewing sensor allows recording for the entire day.

With regards to the sample’s frequency of food selection, we found that the number of home-cooked meals was low, with most meals being either store-bought or restaurant-bought, similar to previous reports [43]. Similarly, a large number (one-third) of the reported drinks contained sugar, with the actual portion potentially being even higher since other drink categories might also have contained added sugar (eg, milk/diary). These dietary habits have been associated with increased body weight [44,45]. Meanwhile, the meal timing across days suggests that Swedish adolescents eat their breakfasts and lunches later during weekends than during weekdays. This time shift is partly corroborated by a previous study in young adults, where the first caloric intake occurred later during weekends, while the last energy intake occurred at similar times during weekdays and weekends [36], potentially due to the de facto effect of the school schedule. Overall, due in part to methodological limitations and differences in definitions, there is currently no consensus regarding the effect of meal timing on health [46], with some studies reporting skipping breakfast may increase the risk of obesity and type 2 diabetes [47,48], while others reporting no such effects [49].

The main strengths of the study were a smartphone app designed to allow easy and accurate real-time meal registration, not relying on recall, and a protocol with no restrictions to participants’ real-life activities. Another strength, based on the results, was the high usage rate of the system. A strength of the system is its modular design, which enables the additional sensors, ensuring high contextual adaptability. In this study, a digital food scale and chewing sensor were used, but when investigating other diseases, such as diabetes, more appropriate sensors could be added, such as a blood glucose monitor.

One limitation of the study was the low number of available study devices, while a limitation for the generalizability of the results was the low number of male participants, which deviates from the Swedish average [50] but is more in line with the sex distribution of the school in question. Another potential limitation was the de facto assumption of 3 main meals per day for compliance estimation, since meal-skipping studies suggest that not everyone eats breakfast, lunch, and dinner [51]. However, the definition of meal skipping in previous studies varies greatly, causing a large range in the frequency of meal skipping (5% to 83%), which provides no reliable baseline for compliance estimation [51]. A potential alternative for measuring compliance would be to ask the user for meal occurrences in the past 30 minutes at random times during the day and then compare these results with spontaneously self-reported meals [36]. This method was avoided due to concerns that additional interaction with the smartphone app may result in reduced compliance. Another protocol limitation was that our study provided additional study smartphones for the app, but this practice was deemed necessary to include iOS users and homogenize the data collection experience. In addition, our reporting system lacked a dedicated category for reporting “noncaloric soft drinks,” which may have resulted in individuals either not reporting those items or reporting it in another category. It should also be noted that the minimalistic reporting approach in our study might introduce additional reporting bias in certain categories. For example, users were able to report milk/dairy-based drinks either in the dedicated category or as “sugary drinks” if they contained sugar. In future iterations of our system, additional user-reporting options can be introduced to resolve this issue (eg, adding the user-selected option to report “added sugar” or “no added sugar” within the milk/diary reports). Finally, one should not ignore the potential observer effect [52], something uniformly affecting the domain of behavioral monitoring, resulting in modification of the observed behaviors due to study participation.

Future studies should aim to repeat the feasibility study by enrolling larger samples and longer data collection periods in order to test the progress of registration compliance over longer periods. In a parallel study in the Netherlands, the chewing sensor received an average wearer comfort score of 3.7 by overweight adults, which is comparable to the score received in the current study [30]. Future studies may benefit from comparing differences in system perception and user compliance between obese and normal-weight individuals. Additionally, based on existing evidence showing that mHealth is more often used by individuals of higher socioeconomic status, additional studies should also aim to include individuals of lower socioeconomic characteristics [53]. Such deployments have a de facto interest for group-based behavioral comparisons but will also evaluate the scalability potential of the system. For validation purposes, the system should be compared to recall methods (eg, 7-day food record and meal habit questionnaires) as well as other automatic recording methods (eg, eButton and Automatic Ingestion Monitor) [41,54]. In practice, the presented methodologies have been the stepping stone for the extension of the data collection and analysis framework in a follow-up research effort [23]. Specifically, our renewed efforts focus on the collection of lifestyle Big Data from children and adolescents, in an effort to create population-level behavioral profiles (eg, meal frequency, food choice frequency), which will then be used by local and national public health authorities as a helpful tool in their efforts to tackle childhood obesity.

In conclusion, no system constraints related to infrastructure and function were identified for deploying the described smartphone-based system in adolescents of medium to high socioeconomic status in Sweden. Also, the identified population profiles regarding the differences in the timing of meals on weekdays vs weekends and the reported frequency of sugary drinks by adolescents provide valuable preliminary information about the dietary habits of the target population. Additionally, these data point towards the usefulness of comparable mHealth systems in providing health-related behavioral information for such populations. Regarding the system use, high school students are well-versed in the use of smartphones, resulting in high acceptability and usability of the smartphone app, with most suggested improvements being related to design rather than functionality. In line with this, the number of participants that required additional information on device use was low. The high registration frequency of main meals indicates the high usability of the system, which, if coupled with appropriate sensors, can facilitate the collection of reliable food intake data. Overall, the system appears promising as a low-effort method to provide accurate measurements of dietary habits, setting the base for future developments of individual-level and group-level mHealth interventions against adolescent obesity.

## Appendix

Multimedia Appendix 1 mHealth evidence reporting and assessment (mERA) checklist.

## Abbreviations

mERA: mHealth evidence reporting and assessment checklist.
mHealth: mobile health.
SUS: System Usability Questionnaire.
