# Supplementary material for: Formative Evaluation of a Smartphone App for Monitoring Daily Meal Distribution and Food Selection in Adolescents: Acceptability and Usability Study
Source: JMIR Mhealth Uhealth. 2020 Jul 21;8(7):e14778. doi: 10.2196/14778 (PMC7404017; doi:10.2196/14778)
Supplement: Multimedia Appendix 1 [file mhealth_v8i7e14778_app1.pdf]

Supplementary material

mHealth evidence reporting and assessment (mERA) checklist

Table 1. Table with information on where to find a response to each criterion of the mERA checklist in the article.

Criteria	Notes	Page no.
1. Infrastructure (population level)	-	6
2. Technology platform	The current software version was only available on Android and was not uploaded to any online store because this was the first iteration of the system.	5
3. Interoperability/ Health information systems (HIS) context	Not applicable. Since the system was in its infancy there was no interoperability or HIS context	-
4. Intervention delivery	Not applicable. In the current study only baseline measurements were made, which are required for a future tailored intervention. The envisioned intervention will provide notifications through the mobile application, based on the baseline measure and the proximity to the set goal.	-
5. Intervention content	The current study was conducted to drive content development.	4
6. Usability/content testing		4
7. User feedback		6
8. Access of individual participants		10
9. Cost assessment		6
10. Adoption inputs/ programme entry		4
11. Limitations for delivery at scale		6
12. Contextual adaptability		10
13. Replicability	The section, with accompanying figures, concerning the Smartphone application is written to increase replicability.	5
14. Data security		5
15. Compliance with national guidelines or regulatory statutes	Not applicable.	-
16. Fidelity of the intervention		7
